# Expression and Surface Display of an Acidic Cold-Active Chitosanase in *Pichia pastoris* Using Multi-Copy Expression and High-Density Cultivation

**DOI:** 10.3390/molecules27030800

**Published:** 2022-01-26

**Authors:** Yanhong Peng, Yaping Wang, Xiaoyan Liu, Ronghua Zhou, Xianqing Liao, Yong Min, Lixin Ma, Ying Wang, Ben Rao

**Affiliations:** 1 National Biopesticide Engineering Technology Research Center, Hubei Biopesticide Engineering Research Center, Hubei Academy of Agricultural Sciences, Biopesticide Branch of Hubei Innovation Centre of Agricultural Science and Technology, Wuhan 430068, China; pengyanhong666@126.com (Y.P.); stwangyaping@163.com (Y.W.); xiaoyanliu6613@163.com (X.L.); zhouronghua1975@126.com (R.Z.); liaoxianqing@nberc.com (X.L.); yong.min@nberc.com (Y.M.); 2 State Key Laboratory of Biocatalysis and Enzyme, Engineering Hubei Collaborative Innovation Center for Green Transformation of Bio-Resources, Hubei Key Laboratory of Industrial Biotechnology, Biology Faculty of Hubei University, Hubei University, Wuhan 430062, China; malixing@hubu.edu.cn

**Keywords:** chitosanase, chiton oligosaccharides, high-density fermentation, multi-copy strains, *Pichia pastoris*, displayed enzyme

## Abstract

Chitosanase hydrolyzes β-(1,4)-linked glycosidic bonds are used in chitosan chains to release oligosaccharide mixtures. Here, we cloned and expressed a cold-adapted chitosanase (CDA, Genbank: MW094131) using multi-copy expression plasmids (CDA1/2/3/4) in *Pichia pastoris*. We identified elevated CDA expression levels in multi-copy strains, with strain PCDA4 selected for high-density fermentation and enzyme-activity studies. The high-density fermentation approach generated a CDA yield of 20014.8 U/mL, with temperature and pH optimization experiments revealing the highest CDA activity at 20 °C and 5.0, respectively. CDA was stable at 10 °C and 20 °C. Thus, CDA could be used at low temperatures. CDA was then displayed on *P. pastoris* using multi-copy expression plasmids. Then, multi-copy strains were constructed and labelled as PCDA(1-3)-AGα1. Further studies showed that the expression of CDA(1-3)-AGα1 in multi-copy strains was increased, and that strain PCDA3-AGα1 was chosen for high-density fermentation and enzyme activity studies. By using a multi-copy expression and high-density fermentation approach, we observed CDA-AGα1 expression yields of 102415 U/g dry cell weight. These data showed that the displayed CDA exhibited improved thermostability and was more stable over wider temperature and pH ranges than free CDA. In addition, displayed CDA could be reused. Thus, the data showed that displaying enzymes on *P. pastoris* may have applications in industrial settings.

## 1. Introduction

Chitin is a linear homopolymer of acetylated β-(1,4)-linked glucosamine residues, is one of the most abundant polysaccharides in the world [1], and is found primarily in insects, crustaceans, nematodes, and fungi. Following deacetylation, chitosan can be generated in a soluble form, suitable for multiple applications. Derived chitosan oligosaccharides are water soluble and exhibit multiple biological functions, including anti-tumor, anti-bacterial, and anti-fungal activities. These features make chitosan and associated oligosaccharides useful in the agriculture, food, and pharmaceutical industries.

Chemical treatment and enzymatic hydrolysis are two primary methods used to derive oligosaccharides from chitosan. However, chemical treatments require large volumes of acids and bases, which are not environmentally friendly [2,3]. Therefore, efforts have been made to derive high efficiency chitosan-degrading enzymes for chitosan oligosaccharide production [4,5,6]. Chitosanase hydrolyzes β-(1,4)-linked glycosidic bonds in chitosan chains to release oligosaccharide mixtures. Chitosanase enzymes are grouped into seven glycoside hydrolase families (i.e., 3, 5, 7, 8, 46, 75, and 80). Families 46, 75, and 80 include most of the known chitosanase enzymes which have been isolated and heterologously expressed, based on their ability to efficiently process chitosan into oligosaccharide mixtures for industrial applications [7,8,9,10,11]. However, polymerization levels in chitooligosaccharide mixtures dynamically change during enzymatic hydrolysis processes. Final hydrophytic mixtures are often (GlcN)2 and (GlcN)3 rich, which means they cannot be used in the food, agriculture and medical industries. To generate chitooligosaccharide mixtures with specific polymerization levels, enzymatic hydrolysis processes have to be stringently regulated. Several methods have been applied (e.g., adding alkali or using high temperature treatments to prevent chitosanase activity), albeit with little success. However, cold-active enzymes have been used under mild reaction conditions to generate different mixtures, suggesting this approach could be valid in producing specific chitooligosaccharide mixtures.

Our lab obtained a *Bacillus glycinifermentans* BT2019 strain with high chitosanase activity at 20 °C. We first cloned the CDA gene (Genbank: MW094131) using a pair of degenerate primers, expressed the cold-active chitosanase using *P.* *pastoris*, and characterized the generated recombinant enzyme. Our analyses showed that CDA converted chitosan to chitooligosaccharides, with different degrees of polymerization, under mild conditions. Then, CDA was surface displayed on *P. pastoris* and used to perform the subsequent reactions. Our data showed that surfaces displaying CDA could also be used to produce specific chitooligosaccharide mixtures. Free and surface displayed CDA were compared and showed potential efficacy for the industrial production of chitosan.

## 2. Materials and Methods

### 2.1. Strains, Reagents and Media

*P. pastoris,* GS115 and *Escherichia coli,* DH5α strains were purchased from Invitrogen (Carlsbad, CA, USA). The pHBM905BDM plasmid was constructed in our laboratory. All culture media, including minimal dextrose (MD,1.34% YNB, 2% dextrose, 4 × 10^−5^% biotin), buffered minimal glycerol (BMGY, 1% yeast extract, 2% peptone, 100 mM potassium phosphate, pH 6.0, 1.34% YNB, 4 × 10^−5^% biotin, 1% glycerol), and buffered minimal methanol (BMMY, 1% yeast extract, 2% peptone, 100 mM potassium phosphate, pH 6.0, 1.34% YNB, 4 × 10^−5^% biotin, 0.5% methanol) were prepared, as described in the *P. pastoris* expression manual (Invitrogen).

### 2.2. Construction of pHBM905BDM Expression Vectors for Free and Surface Displayed CDA Expression

**Figure 1:**
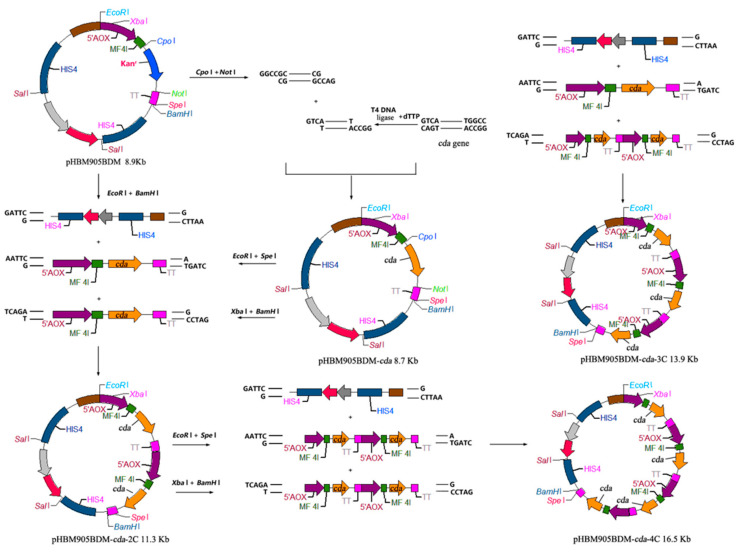
The construction of pHBM905BDM expression vectors.

The CDA sequence (GenBank: MW094131) was modified based on *P. pastoris* codon-usage preference, and synthesized [12] by Sangon BioTech (Shanghai, China). Primers Cdaf/Cdar were synthesized to amplify *CDA,* which was treated by T4 DNA polymerase for 20 min. The PCR product was ligated into *Cpo*I/*Not*I-treated restriction sites of the pHBM905BDM plasmid, and transformed into *E. coli.* The generated pHBM905BDM-CDA plasmid was digested with *Eco*RI/*Spe*I or *Xba*I/*Bam*HI to produce expression cassettes containing the promoter, the leading signal sequence, the target open reading frame and the terminator (Figure 1). pHBM905BDM-CDA1 was further digested with *Eco*RI and *Bam*HI to produce fragments ligated to the expression cassette, to produce pHBM905BDM-CDA2, containing two CDA copies. The method generated recombinant plasmids bearing three and four CDA copies, respectively. The method to verify the multi-copy plasmid included cutting the expression cassette containing the *cda* gene with *Xba*I/*Bam*HI, and then cutting the vector backbone with *Spe*I/*Bam*HI. After double digestion, the size of the products was calculated, and separated on an agarose gel to determine whether the bands were consistent with the calculated products. After confirming the recombinant vectors, they were labelled pHBM905BDM-CDA2, pHBM905BDM-CDA3, and pHBM905BDM-CDA4 [13,14,15].

DNA fragments encoding CDA-AGα1 fusion sequence were synthesized by Sangon BioTech. Using the mentioned method, recombinant expression plasmids were generated containing one, two and three CDA-AGα1 copies. These plasmids were called pHBM905BDM-CDA1-AGα1, pHBM905BDM-CDA2-AGα1, and pHBM905BDM-CDA3-AGα1 [16].

### 2.3. Confirmation of the Number of Copies of the Recombinant Strains Using qPCR

The genomic DNA was extracted from yeast cells using a yeast genomic DNA kit (Omega, New York, NY, USA). The glyceraldehydes-3-phosphate dehydrogenase (*GAP*) gene of *P. pastoris* was used as the reference gene for qPCR. All real-time PCR reactions were performed using the FX96TM real-time PCR Detection System (Bio-rad, Hercules, CA, USA). The program was carried out as follows: 95 °C for 5 min; 40 cycles of denaturation at 95 °C for 30 s; annealing at 58 °C for 30 s; and elongation at 72 °C for 20 s. Fluorescent signal measurements were carried out during each elongation step. Plasmids bearing GAP and the target gene were used to generate the standard curves. The total gene copy numbers of GAP and the target gene in the genomic DNA samples were determined by relating Ct values to the standard curves. The target gene copy numbers that were integrated into the genomes of recombinant *P. pastoris* were calculated based on the ratio of the copy number of the target gene relative to *GAP.*

### 2.4. Expression of CDA and CDA-AGα1 Fusion Proteins

Recombinant *P. pastoris* strains PCDA(1–4)/PCDA(1–3)-AGα1 were cultured in 100 mL BMGY shaking flasks. When the OD_600_ reached 40, cells were harvested and cultured in 50 mL BMMY medium, to which 0.5% methanol was added to induce protein expression every 12 h. When fermentation was terminated, supernatants or cells were harvested by centrifugation at 4 °C and 10,000 g for 5 min. The supernatants were treated and separated by 12% (*w*/*v*) sodium dodecyl-sulfate polyacrylamide gel electrophoresis (SDS-PAGE). The input protein concentrations were determined using the Bradford kit (Beyotime, China) with bovine serum albumin as the standard.

### 2.5. High Density Fermentation of Recombinant Strains

Fed-batch fermentation was performed according to previously described methods. Each recombinant strain, PCDA(1–4)/PCDA(1–3)-AGα1 was cultured in 100 mL flasks containing YEPD medium at 30 °C for 12 h. The seed medium was transferred to 12 L BMGY medium in a 20 L fermenter, and fermentation performed at 28 °C, pH 6.0, under 30% dissolved O_2_. When the optical density reached target density, 1% methanol with PTM1 trace salts were fed to cells at a rate of 3 mL/h/L. Approximately 20 mL cultures were collected every 12 h for enzyme activity assay.

### 2.6. Enzymatic Characterization of Free and Displayed CDA

CDA activity was assayed as follows: Activity was measured at 20 °C in a heterogeneous incubation mixture, containing 50 mg 60% deacetylated chitosan, 1 mL enzyme preparation or harvested cells, and 5 mL 50 mM Tris-HCl buffer (pH 8.5). One activity unit (U) of generated acetic acid was defined as the amount of enzyme required to produce 1 µmol acetic acid per min.

The optimal pH for enzyme activity was determined using different buffering systems (i.e., 0.02 M Na_2_HPO_4_-citric acid (pH 4.0–6.0), sodium phosphate (pH 6.0–7.5), Tris-HCl (pH 7.5–8.8), and glycine-NaOH (pH 8.8–10.6)). To determine the optimal temperature, enzyme activity was measured between 10 °C and 80 °C. Following enzyme incubation at different temperatures for a defined time, remaining enzyme activity was measured to infer thermal stability. The effects of various metal ions and substances on CDA activity were assessed using final 5 mM and 0.5% (*w*/*w*) concentrations, respectively. All experiments were performed in triplicate.

### 2.7. Determination of Kinetic Parameters

The kinetic parameters of the enzymes were determined using colloidal chitosan concentrations of 1 to 10 mg/mL at 20 °C and pH 5.0. Each determination was performed in triplicate. All data were fitted to the Michaelis-Menten equation using non-linear regression to calculate the *Km* and *Vm* [17].

### 2.8. TLC Examination

The supernatants of the chitosan hydrolysate (1 μL) were poured on a silica gel plate. Ethyl acetate: ethanol: water: ammonia (5:5:4:0.3, *v*/*v*) was used as the developing solvent, and 0.1% ninhydrin was used as the chromogenic agent. Color was developed in 10 min at 105 °C.

### 2.9. Assessment of Displayed CDA Using Immunofluorescence and Fluorescence Microscopy

Recombinant cells were labeled for immunofluorescence analysis. Cells were harvested by centrifugation, washed several times in 0.01 M PBS, and resuspended and blocked in PBS containing 1% BSA for 30 min. Then, 1 mL cell suspension was incubated with 5 μL anti-FLAG IgG at 25 °C for 90 min, after which cells were washed and resuspended in PBS containing Alexa FluorTM 488-conjugated goat anti-mouse IgG and incubated for 90 min at 25 °C. Cells were observed under a fluorescence microscope at excitation and emission wavelengths of 488 nm and 510–535 nm, respectively.

## 3. Results and Discussion

### 3.1. Cloning and Identification of the CDA Gene

Chitosanases effectively convert chitosan to chiton oligosaccharides. These latter molecules are widely used in the agriculture, food, chemical, energy, environmental protection, medicine, and other sectors. In considering the wide range of uses of chiton oligosaccharides, scientists have made considerable efforts to generate chitosanases with high conversion efficiencies and stable properties. 

In recent years, our laboratory has cloned several chitosanases. Most enzymes were thermostable, and others were mesophilic enzymes. These enzymes had little or no industrial applications. Thus, we sought to generate a cold-active chitosanase with activities at low temperatures to save energy and precisely control the reaction process. To do this, we used a microbiological selective media approach, where colloidal chitosan was used to screen hundreds of strains to identify a cold-active chitosanase. After extensive screening, we identified a *Bacillus glycinifermentans* BT2019 strain that exhibited the highest enzyme activity at 20 °C. However, this wild strain produced very low levels of chitosanase, with limited industrial application. Therefore, we elevated the expression levels using recombinant protein expression systems. By analyzing genomic information of *B. glycinifermentans* BT2019, a hypothetical protein was identified as chitosanase based on sequence similarities. Based on the protein sequence (WP_048352981.1), we designed a pair of degenerate primers: ATGAARATHCGNCTNAARAARA and HTANTGYTTYAANGGYATRAGR. Using this pair of primers, we amplified a DNA fragment from *Bacillus glycinifermentans* BT2019 and sequence alignments revealed that it encoded a novel chitosanase. The nucleotide sequence of CDA was deposited in GenBank and assigned the accession number, MW094131.

The full-length CDA gene had an open reading frame of 834 base pairs (bp), encoding 278 amino acids, with a theoretical molecular mass of 32 kDa. A signal peptide of 36 amino acids was predicted using SignalP analysis. BLAST results showed that CDA shared 25% identity with a chitosanase from *Streptomyces* N174 [18], and 79% identity with a chitosanase from *Bacillus nakamurai* (KXZ20106.1).

### 3.2. Construction of Recombinant Strains

Previous studies showed that a cold-active chitosanase was expressed in *E. coli* and converted chitosan into chitooligosaccharides, with different levels of polymerization [19]. However, no related studies have been reported to have produced cold-active chitosanases using *P. pastoris*. Here, a *P. pastoris* multi-copy expression system and high-density fermentation have been used for other enzyme processes in our laboratory. Thus for the first time, we demonstrate effective expression and evaluation of CDA in *P. pastoris,* with potential for industrial applications.

**Figure 2:**
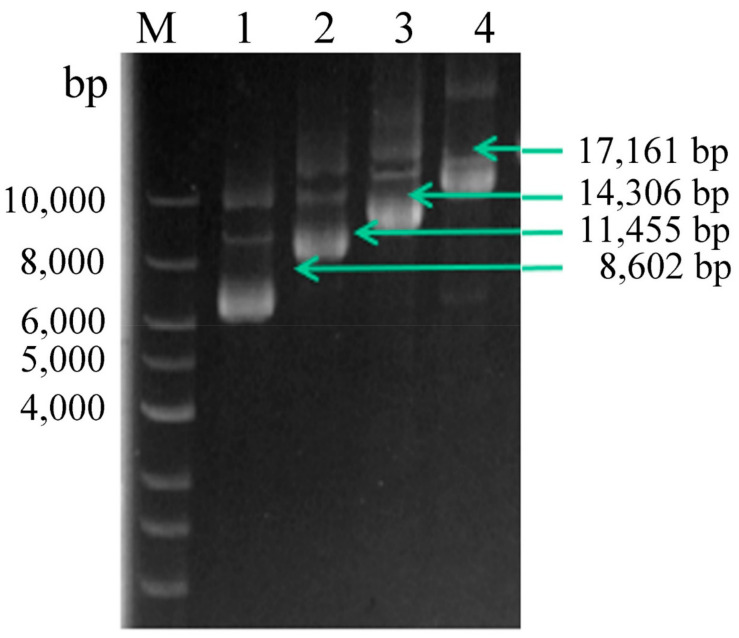
Multi-copy plasmid verification. Lane M, DNA Marker; lane 1, linearized pHBM905BDM-CDA1; lane 2, linearized pHBM905BDM-CDA2; lane 3, linearized pHBM905BDM-CDA3; lane 4, linearized pHBM905BDM-CDA4.

To improve CDA expression levels, expression plasmids harboring multiple copies of *CDA* were constructed. The multi-copy plasmids were identified using agarose gel electrophoresis (Figure 2). Then, they were confirmed using enzyme-digestion, as described in Section 2.2 (data not shown). *P. pastoris* GS115 cells were transformed with the recombinant plasmids, resulting in the recombinant strains, PCDA1, PCDA2, PCDA3, and PCDA4. In addition, plasmid pHBM905BDM was also transformed into GS115, generating the control strain, PCCC.

**Figure 3:**
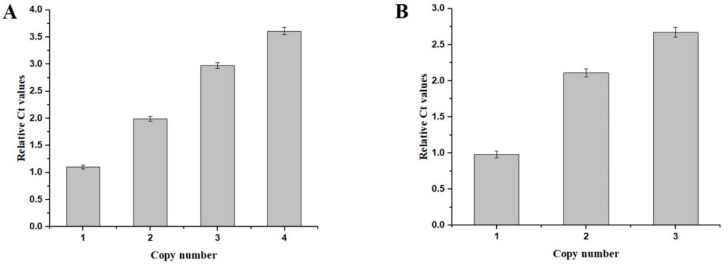
qPCR-based evaluations of the copy numbers of the recombinant strains. (**A**) qPCR of the CDA expression cassettes integrated into the host strains. (**B**) qPCR of the CDA-AGα 1 expression cassettes integrated in the host strains.

qPCR was performed as described in Section 2.3. We confirmed the *CDA* copy number in recombinant strains; standard curves for *CDA* and *GAP* were generated, showing the correlations between Ct values and copy numbers. The *CDA* Ct values of strains PCDA1, PCDA2, PCDA3, and PCDA4 were 1.1, 1.99, 2.98, and 3.61 fold higher than *GAP*, respectively (Figure 3), thus confirming that *CDA* was present in multiple copies in our strains.

**Figure 4:**
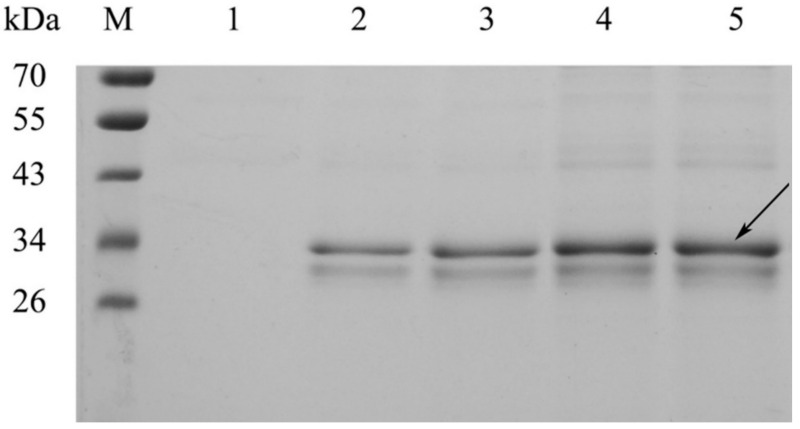
SDS-PAGE analysis of CDA secreted into cell culture supernatants. M, protein molecular weight marker. Lanes 1–4, PCDA1, PCDA2, PCDA3, and PCDA4, respectively.

Strains were cultured, and after four days of induction using methanol, and reaching an OD_600_ of ~40, strain supernatants were subject to SDS-PAGE and enzyme activity analysis. SDS-PAGE (Figure 4) revealed that all supernatant samples contained a band of 32 kDa, with band intensities increasing with an increasing CDA copy number. Enzyme activity analyses showed that high copy CDA strains produced greater enzyme levels (Table 1). Enzyme activities for PCDA2, PCDA3, and PCDA4 were 1.88, 2.92, and 3.86 times higher than that of PCDA1, which were lower than the theoretical value of the gene dosage. This was because elevated protein expression levels influenced host strain metabolism. We previously used the pHBM905BDM plasmid to produce several recombinant enzymes, including amino acid oxidase, glutamate oxidase and a thermostable chitosanase [20,21]. This reagent effectively elevated recombinant protein expression levels via the construction of multi-copy plasmids. In this study, this strategy was used to CDA expression, demonstrating that increased gene copy numbers improved CDA yields. As PCDA4 produced the highest CDA levels, it was chosen for subsequent experiments.

### 3.3. High Density Fermentation Using the PCDA4 Strain

**Figure 5:**
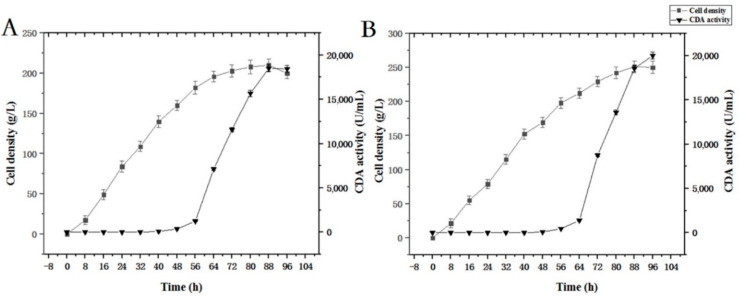
Time course of free CDA fermentation. (**A**) Induction was initiated when OD_600_ = 150; (**B**) induction was initiated when OD_600_ = 200.

We evaluated PCDA4 high-density fermentation to assess CDA expression levels according to fermentation strategy according to the Section 2.5 (Figure 5). First, methanol was added to induce CDA production when the cell density reached 150 g/L. After fermentation, cell density increased from 150 g/L to 210 g/L, generating a 4.7-fold increase relative to shake-flask fermentation. CDA enzyme activity reached 18512.6 U/mL, which was a 71-fold increase when compared with shake-flask data. Then, protein induction was initiated by adding methanol when the cell density reached 200 g/L. After fermentation, final cell density and enzyme activity reached 250 g/L and 20014.8 U/mL, respectively. This represented a 5.9- and a 77-fold increase, respectively. According to these results, an induction density of 200 g/L was optimal, and was used for subsequent fermentation processes. Under fed batch conditions, more oxygen and methanol were provided to the yeast cells than in flasks. Thus, the expression level per cell was improved. Based on our multi-copy expression strategy, high density fermentation further improved cell density and CDA enzyme activity when compared to shake-flasks.

### 3.4. CDA Characterization

**Figure 6:**
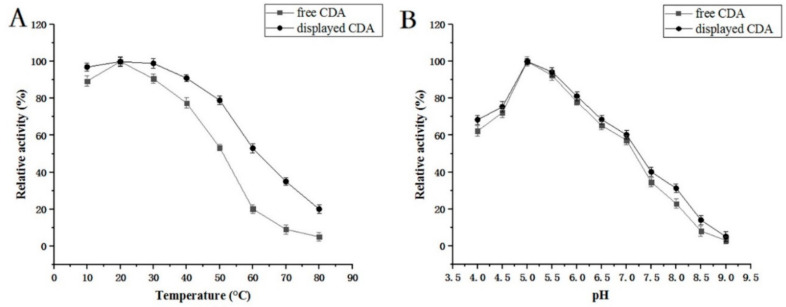
(**A**) Temperature optimization for CDA enzyme activity. Enzyme activity at 30 °C was set to 100%; (**B**) pH optimization for CDA enzyme activity. Enzyme activity at pH 5.0 was set to 100%.

CDA activity was measured at 10, 20, 30, 40, 50, 60, 70, and 80 °C and a pH range of 3, 4, 5, 6, 7, 8, and 9 (Figure 6). Our data showed that maximum CDA activity occurred at 20 °C. When the temperature >70 °C, activity was lost. Furthermore, the optimal pH was 5, while 70% of CDA activity was retained between pH 4 and 6.5. All CDA activity was lost when the pH was >8.5.

**Figure 7:**
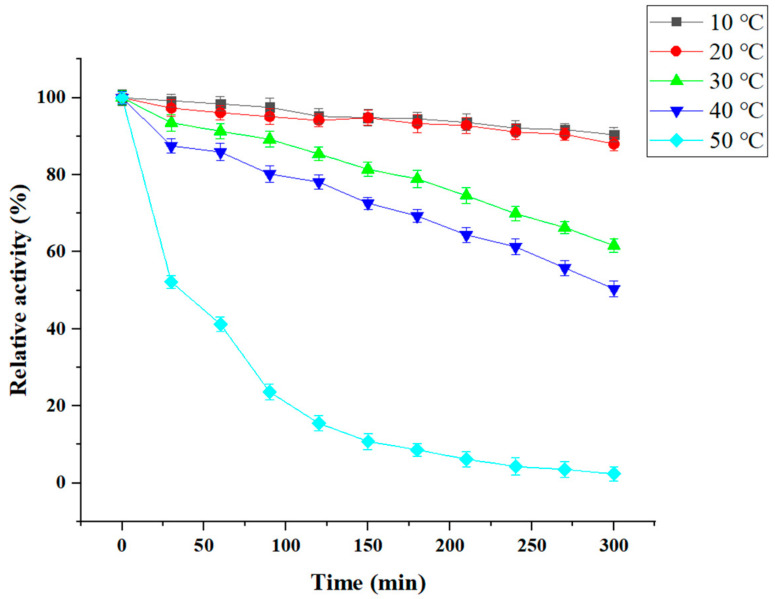
Temperature stability of CDA. The initial enzyme activity was set to 100%.

We next studied CDA thermostability at different temperatures, including 10, 20, 30, 40, and 50 °C for different times (Figure 7). Our data showed that 95% of CDA activity was maintained after the enzyme was incubated at 10 °C for 120 min, and almost 91% activity remained after 300 min. At 30 °C, 85% of CDA activity remained after incubation for 150 min. At 50 °C, CDA activity diminished rapidly (e.g., 15% of CDA activity was maintained for 120 min, and was lost after 270 min). This observation demonstrated that *P. pastoris* CDA exhibited good cold-active properties. 

We investigated the influence of metal ions on CDA expression (Table 2). Our data demonstrated that Mg^2+^ significantly improved CDA activity by approximately 20%. However, Mn^2+^ restrain CDA activity by 98%. Other metal ions, such as Cu^2+^, and Fe^2+^ inhibited CDA activity by 60% and 55%, respectively. Accordingly, Mg^2+^ was added to reaction mixtures to accelerate chitooligosaccharide formation.

We also investigated CDA kinetic parameters. Under conditions described in the Materials and Methods Section 2.7, the Michaelis constant (*Km*) was 1.92 ± 0.07 mg/mL, and the Vmax reaction rate was 395.65 ± 7.34 µmol/min. 

During chitosan hydrolysis reactions, the pH of the chitosan reaction mixture must be low. Indeed, chitosan substrates cannot dissolve in high pH buffers (pH > 5.5), as such mixtures cannot be hydrolyzed by chitosanase. However, the optimal pH of many chitosanases is >5.5. For example, the optimal pH of several reported chitosanases (i.e., CsnW2 from *Aspergillus* sp. W-2 and MsChoA (pH 5.5) from *Mitsuaria* sp. 141) are >5.5 [9,11,19]. Thus, these enzymes are unsuitable for chitosan reactions, and only those enzymes with the highest activity under acidic conditions (<5.5) have potential industrial applications. In general, most of the normal enzymes exhibit optimal activities at temperatures of 30–60 °C. In the past, researchers preferred thermo-chitosanases because high temperatures accelerated reactions, thereby preventing contamination and decreasing reaction mixture viscosity. However, in recent years, cold-active enzymes have attracted greater attention, because lower reaction temperatures save on energy consumption, with more controlled chitooloigosaccharide production. Our cloned CDA proved to be an acidic (pH 5) cold-active (20 °C) chitosanase. Thus, these key characteristics made it a good candidate for controlled chitooloigosaccharide production (next section).

### 3.5. Large-Scale Hydrolysis and Analysis of Chitosan Oligosaccharides with TLC

**Figure 8:**
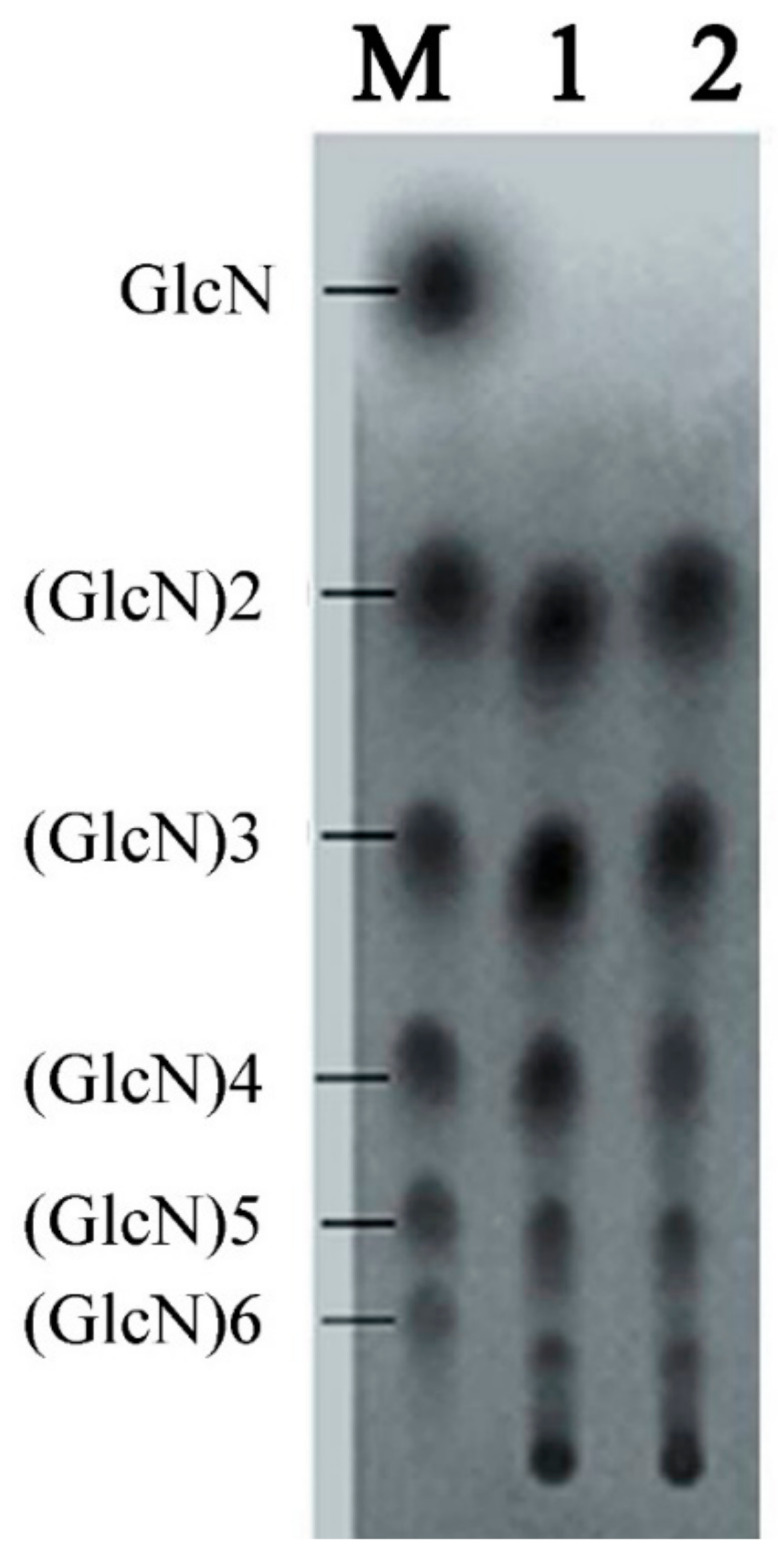
TLC analysis of the products from a large-scale hydrolysis study using free CDA. Lane M, standard mixture of chitosan oligosaccharides; lane 1, hydrolysis products of chitosan for 24 h; lane 2, hydrolysis products of chitosan for 36 h.

We suspended 10 kg chitosan powder in 100 L water, 50 L acetic acid, and 1 L of CDA supernatant from high density fermentation. The reaction was performed three times at 20 °C for 24 and 36 h. Reactions were terminated by incubation at 80 °C for 1 h. We then performed TLC to detect chitosan products. Our results showed that (GlcN)2 to (GlcN)6 were the main products for reactions performed for 24 h (Figure 8). For 36-h reactions, the hydrolytic products were mainly (GlcN)2, (GlcN)3, (GlcN)4, and (GlcN)5. Therefore, performing reactions at 24 h generated optimal polymerization production. 

Several studies have shown that monosaccharides and disaccharides have low biological activities [1,2,3]. Chitosan oligosaccharides, with a degree of polymerization of 2–6, exhibit significant antibiotic effects, which enhance protection against microbial infections in mice, and increase the human body’s resistance. The molecules also appear to regulate the production of plant rhizobia, and activate plant resistance against pathogenic microorganisms. These shell oligosaccharides also prevent constipation, heart disease, induce weight loss, reduce LDL levels, promote calcium absorption, improve health, and enhance immunity and other functions. Thus, prepared chitosan oligosaccharides are vitally important to several commercial sectors. However, many chitosanase exo-chitosanase activity; the generated chitosan oligosaccharide mixtures consist mainly of GlcN and (GlcN)2, with no biological activity.

Using CDA, we generated chitosan oligosaccharide mixtures mainly containing (GlcN)2, (GlcN)3, (GlcN)4, (GlcN)5, and (GlcN)6. Therefore, this enzymatic reaction can be applied to the industrialized production of oligochitosan products. 

### 3.6. Construction and Expression of a CDA Surface Display System in P. pastoris

Yeast cell surface display systems are widely used to display foreign proteins [16]. Here, we tried to display CDA on the surface of *P. pastoris.* For this, we used the N-terminal fusion display α-agglutinin from *S. cerevisiae* as the anchor protein to *P. pastoris*. A fusion fragment containing *CDA*, a Gly-Gly-Gly-Ser sequence, a FLAG tag, and the Agα1 domain from *S. cerevisiae* was synthesized. We then constructed multi-copy expression plasmids of these fusion genes. These plasmids contained one, two, and three copies of the *CDA*-Agα1 fusion gene; pHBM905BMD-CDA1-AGα1, pHBM905BMD-CDA2-AGα1, and pHBM905BMD-CDA3-AGα1, respectively. Plasmids were then electro-transformed into *P. pastoris* to produce the recombinant strains, PCDA1-AGα1, PCDA2-AGα1, and PCDA3-AGα1, respectively. qPCR confirmed that each strain contained the corresponding CDA-AGα1 copy numbers (Figure 3B). 

Recombinant strains were cultured and induced in shake-flasks, the cells were harvested, and CDA activity was assessed. Our data showed that biomass densities from multi-copy strains were similar; however, the strain carrying three CDA1-Agα1 copies showed the highest CDA activity (Table 3). When compared with the other shake-flask cultures, the enzyme activities of PCDA2-Agα1 and PCDA3-Agα1 were 1.75 and 2.2-fold higher than PCDA1-Agα1. We therefore used PCDA3-Agα1 for subsequent high-density fermentation. Being consistent with free CDA expression, multi-copy expression approach could also improve total displayed CDA activity. This was the first time an in vitro multi-copy expression system was applied to chitosanase surface display.

As free enzymes are unstable and cannot be used repeatedly, it was inconvenient to separate the enzyme and the reaction product. Thus, enzymes were immobilized using several methods (e.g., surface display on yeast). This method enhanced protein affinity and thermostability. Here, we applied the in vitro multi-copy expression approach to display chitosanase on the cell surface, demonstrating improvements in surface display productivity.

### 3.7. Detection of Displayed CDA Using Immunofluorescence and Fluorescence Microscopy

**Figure 9:**
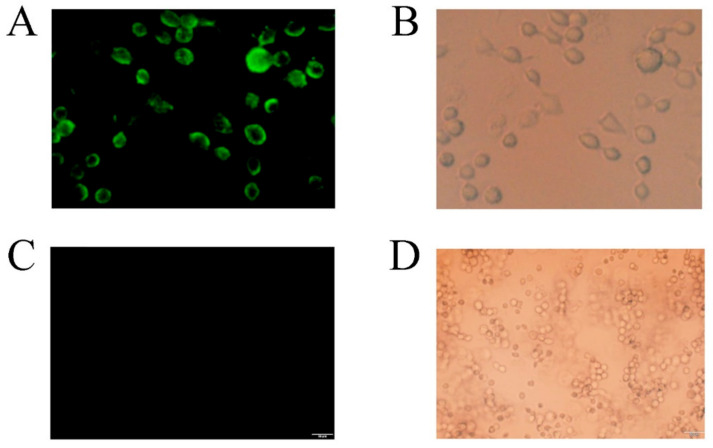
Laser confocal analysis of recombinant cells (displayed CDA) and control cells. Recombinant cells (displayed CDA): (**A**) the excitation source was turned on. Fluorescent cells indicate that CDA was successfully surface displayed on yeast; (**B**) the natural light source was turned on. Control cells: (**C**) the excitation source was turned on, and (**D**) the natural light source was turned on.

We used immunofluorescence to detect CDA on the yeast cell surface. Figure 9 revealed fluorescence (Alexa FluorTM 488 excited) on the cell surface of PCDA3-AGα 1, but not control cells (*P. pastoris* transformed with pHBM905BMD-AGα1). Strains PCDA1-AGα1 and PCDA2-AGα1 were also subjected to immunofluorescence, and showed fluorescence on the yeast surface (data not shown). These data indicated that CDA was successfully displayed on the *P. pastoris* surface.

### 3.8. High Cell Density Fermentation of Strains Expressing Recombinant Surface Displayed CDA

**Figure 10:**
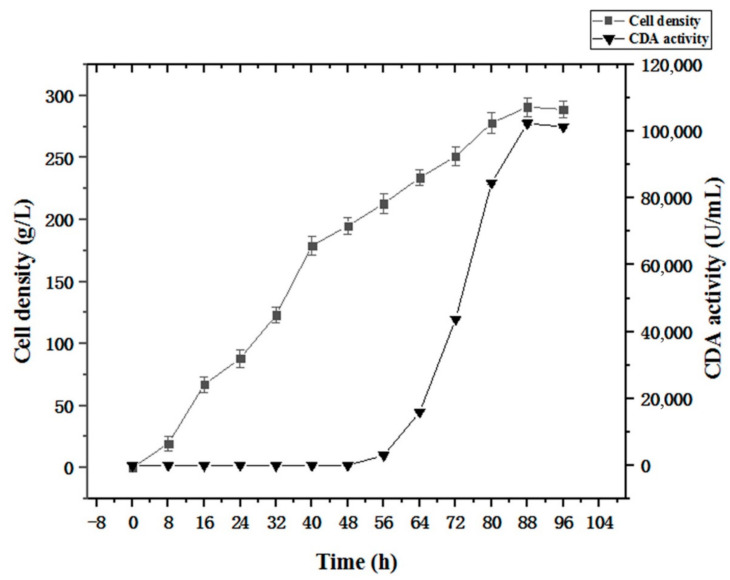
Time course of displayed CDA fermentations based on a fermentation strategy described in the Section 2.5.

The fermentation strategy (Section 2.5) was applied to recombinant strain PCDA3-AGα1 fermentation (Figure 10). When the cell density reached 200 g/L after glycerol feeding periods, methanol was added to induce CDA-AGα1 expression. The cell density increased to 290 g/L, which was a 4.8-fold increase over shake-flasks. CDA activity reached 102415 U/g dry cell weight after 96 h of fermentation, which was a 1.6-fold increase over shake-flasks. Thus, high cell density fermentation markedly improved cell densities and CDA activity, when compared to shake-flasks. Therefore, combining multi-copy expression strains and high-density fermentation significantly improved displayed CDA productivity.

### 3.9. Displayed CDA Characterization on the P. pastoris Cell Surface

We examined the displayed CDA features. Our data indicated that most features were the same as that of free CDA, however, displayed CDA was more stable (Figure 5 and Figure 6); e.g., the displayed CDA retained 90% of its maximum activity at temperatures 20–40 °C, and pH 4–8. This activity decreased rapidly when the temperature was >50 °C or pH > 8. Displayed CDA thermostability was better than free CDA. It retained 90% initial activity for 300 min at 20–40 °C; but 60% of activity was lost when temperatures >50 °C. When displayed CDA was incubated at 60 °C above for 30 min, its activity was mostly lost. These data revealed that displayed CDA showed improved thermostability, and was stable over wider temperature and pH ranges, than free CDA.

### 3.10. Recycling of Displayed CDA and Conversion of Chitosan into Different Chitooligosaccharides

**Figure 11:**
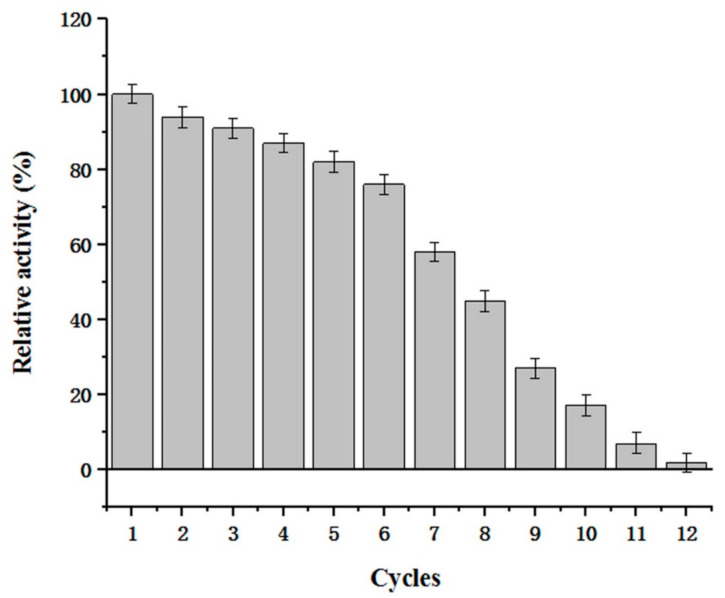
Reusability of displayed CDA. The remaining activity represents the displayed CDA activity after being used for N cycles/unused displayed CDA activity.

By using displayed CDA, we successfully converted 10 kg of chitosan (100 L water, 50 L acetic acid, and 100 g supernatant CDA) into chitooligosaccharide mixtures in 24 h. The experiments revealed that (GlcN)2–(GlcN)6 were the main reaction products over this timeframe (data not shown) and performing reactions over this timeframe generated optimal polymerization products. In addition, as surface display enzymes may be used several times, the recycling efficiency was evaluated (Figure 11). Our data showed that displayed CDA activity remained high after reactions were performed several times. For example, ~76% displayed CDA activity was retained after six reaction cycles. Displayed CDA can be used several times to produce chitooligosaccharide mixtures with different polymerization levels. Additionally, displayed CDA facilitated easy purification of chitooligosaccharide products. The displayed and free CDA characterizations were similar, but the displayed CDA could be reused more frequently. Thus, in future studies and industrialization application methods, displayed CDA should be considered instead of free CDA.

## 4. Conclusions

We present data on a cold-adapted chitosanase application for chitooligosaccharide mixture production. CDA was expressed as free and surface-displayed in *P. pistoris*. Multi-copy expression and high cell density fermentations were used in a combinatorial approach to assess free and surface-displayed CDA expression levels. We achieved free CDA expression yields of 20014.8 U/mL and displayed CDA expression yields of 102415 U/g dry cell weight. Free and displayed CDA may be used to produce chitooligosaccharide mixtures with different polymerization levels, for different purposes. At 20 °C for 24 h, chitin was converted to *chitooligosaccharide* mixtures, primarily consisting of (GlcN)2–(GlcN)6, which are suitable for industrialization.

## Figures and Tables

**Table 1:** Comparisons of the enzyme activity of PCDA1, PCDA2, PCDA3, and PCDA4.

Strain	Final Cell Density (g/L)	Protein Concentration (mg/mL)	Enzyme Activity (U/mL)
PCDA1	46.2 ± 1.22	0.029 ± 0.002	66.8 ± 5
PCDA2	43.1 ± 1.31	0.048 ± 0.015	125.6 ± 6
PCDA3	43.4 ± 1.18	0.075 ± 0.046	195.1 ± 2.5
PCDA4	42.2 ± 1.15	0.121 ± 0.096	258.5 ± 2.1

**Table 2:** The effect of metal salts and chemical agents on the activity of free CDA.

Metal Ion	Concentration	Relative Activity (%)
No addition	0 mM	100
Fe^2+^	5 mM	43.9
Cu^2+^	5 mM	35.9
Mg^2+^	5 mM	121.2
Ca^2+^	5 mM	115.1
Mn^2+^	5 mM	98.5
Co^2+^	5 mM	21.5
Cr^2+^	5 mM	11.4
Zn^2+^	5 mM	8.9
Fe^3+^	5 mM	6.5

**Table 3:** Comparations of the enzyme activity of PCDA1- AGα1, PCDA2-AGα1, and PCDA3-AGα1.

Strain	Final Cell Density (g/L)	Enzyme Activity (U/g)
PCDA1- AGα1	58.5 ± 2.21	29012.2 ± 3.21
PCDA2-AGα1	57.3 ± 2.13	50810.8 ± 3.12
PCDA3-AGα1	59.9 ± 2.21	64112.1 ± 3.25

## Data Availability

Not applicable.

## Sample Availability

Samples of the compounds are available from the authors.

## Abbreviations

CDA	cold-adapted chitosanase
GlcN	2-amino-2-deoxy-D-glucose
GAPDH	glyceraldehyde-3-phosphate dehydrogenase
MD	minimal dextrose
BMGY	buffered minimal glycerol
BMMY	buffered minimal methanol
